# Thermo-Mechanical Properties and Phase-Separated Morphology of Warm-Mix Epoxy Asphalt Binders with Different Epoxy Resin Concentrations

**DOI:** 10.3390/molecules29143251

**Published:** 2024-07-09

**Authors:** Chengwei Wu, Haocheng Yang, Xinpeng Cui, Jun Cai, Zuanru Yuan, Junsheng Zhang, Hongfeng Xie

**Affiliations:** 1 MOE Key Laboratory of High Performance Polymer Materials and Technology, School of Chemistry and Chemical Engineering, Nanjing University, Nanjing 210093, China; 522022240039@smail.nju.edu.cn (C.W.); 522023240043@smail.nju.edu.cn (H.Y.); 15299908527@163.com (X.C.); 2 Public Instrument Center, School of Chemistry and Chemical Engineering, Nanjing University, Nanjing 210023, China; caijun@nju.edu.cn; 3 Modern Analysis Center, Nanjing University, Nanjing 210023, China; zryuan@nju.edu.cn

**Keywords:** epoxy asphalt, phase separation, mechanical properties, glass transition temperature, confocal microscopy

## Abstract

The performance and phase-separated microstructures of epoxy asphalt binders greatly depend on the concentration of epoxy resin or bitumen. In this paper, the effect of the epoxy resin (ER) concentration (10–90%) on the viscosity, thermo-mechanical properties, and phase-separated morphology of warm-mix epoxy asphalt binders (WEABs) was investigated using the Brookfield rotational viscometer, differential scanning calorimetry (DSC), dynamic mechanical analysis (DMA) and laser scanning confocal microscopy (LSCM). Due to the high reactivity of epoxy resin, the viscosity of WEABs increases with time. Furthermore, the initial viscosity of WEABs decreases with the ER concentration. Depending on the ER concentration, the viscosity–time behavior of WEABs is divided into three stages: slow (10–40%), fast (50–80%), and extremely slow (90%). In the slow stage, the viscosity slightly increases with the ER concentration, while the fast stage shows an opposite trend. DSC and DMA results reveal that WEABs with 10–80% ER exhibit two glass transition temperatures (T_g_s) for cured epoxy resin and bitumen. Moreover, the T_g_s of epoxy resin and bitumen increase with the ER concentration. However, WEAB with 90 % ER has only one T_g_. LSCM observation shows that phase separation occurs in all WEABs. For WEABs containing 10–40% ER, spherical epoxy particles act as the discontinuous phase and disperse in the continuous bitumen phase. However, in WEABs with 50–90% ER, phase inversion takes place. Contrarily, bitumen particles disperse in the continuous epoxy phase. The damping properties of WEABs with the continuous epoxy phases increase with the ER concentration, while the crosslinking density shows an opposite trend. The occurrence of phase inversion results in a sharp increase in the tensile strength of WEABs. For WEABs with the continuous epoxy phases, the elongation at break increases with the ER concentration. The toughness first increases and then decreases with the ER concentration. A maximum toughness value shows at 70% ER.

## 1. Introduction

Epoxy asphalt, thermosetting polymer-modified asphalt, was developed over 60 years ago and is widely used in airfield and bridge construction [1]. Epoxy asphalt was first developed in the United States of America (USA) in the late 1950s, which was used as the surface course of San Mateo-Hayward Bridge in 1967 and showed an extremely good performance and lasted for almost 48 years [2,3]. Since 1967, many steel bridges, including San Francisco-Oakland Bay Bridge and Golden Gate Bridge, have been paved using epoxy asphalt materials in the USA, Canada, Brazil, and China. Especially in China, epoxy asphalt has become the main paving material for the construction of steel bridges and has been utilized on hundreds of long-span steel bridges since 2001 [4]. Based on its usage for bridge construction, epoxy asphalt is classified into two catalogs: bond (tack) coat and binder [5]. The binder is used to bond the aggregates to produce epoxy asphalt concrete (EAC), while the bond coat is used to bond the steel deck plate and concrete layer or two concrete layers [6,7]. 

Epoxy asphalt binders (EABs) are cataloged into three groups depending on the mixing temperature: cold-mix (ambient temperature), warm-mix (110–130 °C), and hot-mix (160–190 °C) [8,9,10,11]. Among all these binders, warm-mix epoxy asphalt binder (WEAB) and hot-mix epoxy asphalt binder (HEAB) are the most used ones [12]. Apart from mixing temperature, these two binders are quite different due to their different curing agent systems [13,14,15,16]: (1) HEAB has an extremely longer allowable construction time (over 150 min) than WEAB (57–94 min). (2) HEAB is composed of bitumen (asphalt binder), epoxy oligomer and curing agent, while WEAB has only two components: the mixture of curing agent and bitumen and epoxy oligomer stored in different drums. In this case, heating equipment is needed to heat the drum to release the mixture of curing agent and bitumen. (3) After compaction, HEAB needs 5–10 days to complete the cure reaction at ambient temperature, whereas 30–45 days are required for WEAB. In this circumstance, old bridges are hard to maintain with WEAB. (4) WEAB needs special equipment, such as a metering device, to complete the mixing, which unavoidably increases the construction difficulty. For HEAB, the epoxy oligomer and curing agents of epoxy resin (ER) are pre-weighted in two 20 L barrels. After heating at 60 °C, the two barrels are mixed directly. Afterward, the mixture is transported and mixed with bitumen. (5) Due to its lower mixing temperature, WEAB is more environmentally friendly than HEAB. (6) WEAB is used to produce the concrete in combination with the epoxy asphalt bond coat, while HEAB is applied with a B-staged epoxy bond coat [17,18].

As noted above, the composition of epoxy asphalt primarily includes bitumen and epoxy resin, which is made up of epoxy oligomers or monomers, curing agents, and other modifying ingredients, such as diluents, tougheners, and fillers [19,20,21,22]. In this case, the properties and phase-separated microstructures of EABs significantly depend on the concentration and composition of bitumen and ER. Zeng et al. studied the morphology and properties of HEABs containing 10–50% epoxy resin [23]. When the epoxy loading increases to a mass fraction of 37%, phase invention occurs. That is to say, the originally continuous bitumen phase converts into a continuous epoxy phase, which results in a dramatic improvement in the mechanical properties. For hot-mix epoxy asphalt concrete (HEAC), the Marshall stability, dynamic stability, bending strength, and fracture energy increase with the epoxy resin concentration. However, the flow value exhibits a contrary trend. Luo’s groups investigated the performance and morphology of HEABs with a wider range of epoxy resin concentrations (10–90%) [24,25,26]. The tensile strength of HEABs decreases with the increase in the epoxy resin concentration, while the elongation at break exhibits an opposite trend [24,25]. At the same temperature, the viscosity of HEABs with 10–50% epoxy resin increases with the epoxy resin concentration. The dynamic stability, maximum bending strain, and freeze–thaw splitting strength ratio of HEACs with 30–50% epoxy resin increase with the epoxy resin concentration [25]. Fluorescence microscopy observations revealed that bitumen is the continuous phase when the epoxy resin concentrations of HEABs are 10–30%. At a 40% epoxy resin concentration, the co-continuous phase morphology forms, i.e., both bitumen and cured epoxy resin exhibit continuous structures. With the further increase in the epoxy resin concentration, phase inversion occurs, which dramatically enhances the mechanical properties of HEAB due to the formation of the continuous epoxy phase [26,27]. 

The performance–morphology relationship of HEABs with different epoxy resin concentrations has been well investigated, as discussed above. However, little attention has been paid to WEABs, which have different epoxy resin systems and reaction rates in comparison to HEABs. In this paper, the viscosity vs. temperature, glass transition temperature (T_g_), storage modulus, damping properties, Cole–Cole plots, mechanical properties, and phase-separated microstructures of WEABs containing 10–90% epoxy resin were studied by Brookfield rotational viscometer, differential scanning calorimetry (DSC), dynamic mechanical analysis (DMA), and laser scanning confocal microscopy (LSCM). Furthermore, the viscosity characteristic at 120 °C, T_g_s of WEABs and phase-separated morphology of WEABs were compared to those of the neat bitumen and pure epoxy resin.

## 2. Materials and Methods

### 2.1. Materials

Asphalt binder (bitumen) with a penetration grade of 60/80 was provided by China Offshore Bitumen (Taizhou) Co., Ltd. (Taizhou, China). Table 1 summarizes the overview of the binder. Epoxy oligomer with epoxide number of 0.52 mol/100 g was procured from Nantong Xingchen Synthetic Material Co., Ltd. (Nantong, China). Self-prepared acid was used as the curing agent. 

### 2.2. Preparation of WEAB

The bitumen preheated at 150 °C was mixed with the curing agent in a beaker at 120 °C and 200 min^−1^ for 5 min, followed by adding epoxy oligomer and mixing at the same speed for another 2 min. After being poured into a Teflon mold, the uncured WEAB was cured at 120 °C for four hours. The weight ratio of epoxy oligomer to the curing agent is 100:265. The ER content in WEABs increases from weight ratios of 10% to 90% with a 10% increase interval. Correspondingly, WEABs are named as EA10, EA20, EA30, EA40, EA50, EA60, EA70, EA80, and EA90, respectively.

### 2.3. Methods

#### 2.3.1. LSCM

The phase-separated morphology of WEABs was studied using a Carl Zeiss LSM 710 LSCM instrument (Jena, Germany). The laser source was an Ar+ laser light of 488 nm. The samples for observation were prepared as follows: a drop of uncured WEAB liquid was dripped onto a sliding glass and covered by a cover glass, followed by being cured at the same temperature and time as mentioned in Section 2.2.

Three LSCM images with ×100 magnification were employed to evaluate the average diameters of discontinuous domains. In addition, average diameters can be calculated by the following equations [6]:

(1) $$d_{n}=\frac{\Sigma n_{i}d_{i}}{\Sigma n_{i}}$$

(2) $$d_{w}=\frac{\Sigma n_{i}d_{i}{\;\;}^{2}}{\Sigma n_{i}d_{i}}$$

where *d_i_* is the diameter of the *n_i_* particle.

#### 2.3.2. DSC

The Perkin-Elmer Pyris 1 differential scanning calorimeter (Norwalk, CT, USA) was used to determine the T_g_s of WEABs under the protection of argon flow (20 mL/min). The cured WEAB in an aluminum crucible was heated at a ramping rate of 20 K/min from −50 to 100 °C and held at 100 °C for 2 min to eliminate the thermal history. Then, the sample was rapidly cooled to −50 °C, followed by the second heating run at a ramping rate of 20 K/min from −50 to 100 °C. The second heating run was used to determine the T_g_ of WEAB.

#### 2.3.3. DMA

The viscoelastic performance of WEABs was evaluated by a DMA + 450 DMA instrument (01 dBMetravib, Limonest, France). The measurement was conducted on the tension mode. A sample with the size of 20 × 20 × 3 mm^3^ was heated from −50 to 100 °C at a frequency of 1 Hz and a ramping rate of 3 K/min.

#### 2.3.4. Viscosity–Time Behavior

The viscosity as a function of time for the uncured WEAB during heating at 120 °C was obtained by a No. 28 spindle on a Changji NDJ-2 rotational viscometer (Shanghai, China). The viscosity was recorded every 5 min.

#### 2.3.5. Tensile Properties

Tensile tests were determined by the Instron universal testing machine (Instron 3366, Norwood, MA, USA) as per ASTM D638. The load cell was 10 kN. The sample was tested at a crosshead speed of 200 mm/min. Five duplicates were measured to procure the average values.

## 3. Results and Discussion

### 3.1. Viscosity versus Time

**Figure 1:**
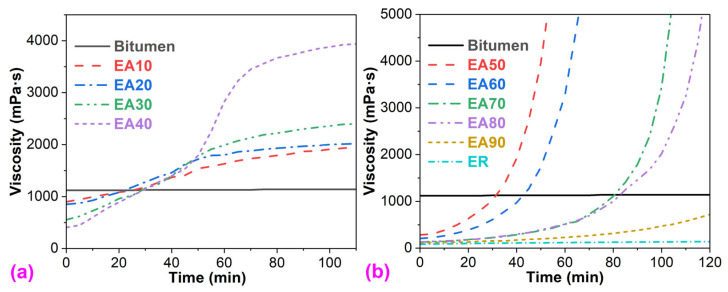
Viscosity–time curves of bitumen, epoxy resin, and WEABs at 120 °C: (**a**) 10–40% ER and (**b**) 50–90% ER and pure ER.

It is well acknowledged that the viscosity of bitumen is only temperature-dependent, which declines with the increase in the temperature. At the same temperature, the viscosity of the binder is almost constant (Figure 1). However, the viscosity of epoxy asphalt binders is dependent on both the temperature and time due to the reactive feature of epoxy resin, which releases heat and results in an increase in epoxy molecular weight [12]. Thus, apart from the temperature, the viscosity of epoxy asphalt binders increases with time (Figure 1). However, the influence of the temperature on the viscosity relies on the kind of epoxy asphalt binders [5]. In other words, epoxy asphalts with different epoxy resin systems have various temperature-dependent behaviors. With the increase in temperature, WEAB’s viscosity increases, whereas HEAB’s viscosity shows an opposite trend [12].

The viscosity–time behavior of WEABs with different ER concentrations shows three stages. In the first stage (10–40% ER), the viscosity of WEABs steadily increases with time (Figure 1a). For the WEAB with 40% ER, a sharp increase in viscosity occurs in the range of 50–70 min. After 70 min, the viscosity continues to increase steadily. In the second stage (50–80% ER), a dramatic increase in viscosity occurs (Figure 1b), which is caused by the phase inversion in WEABs, as will be discussed in Section 3.7. Furthermore, the sharp increment trend of viscosity decreases with the ER concentration. At 90% ER concentration, WEAB’s viscosity increases very slowly with time, whose value is only greater than that of the pure ER at a certain time, and much lower than those of other WEABs and the neat bitumen, as shown in Figure 1.

**Figure 2:**
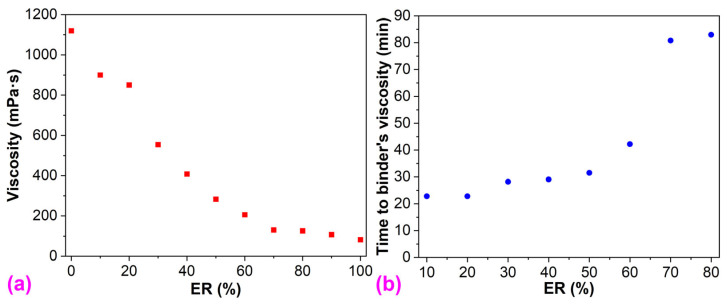
(**a**) Initial viscosity of the bitumen, pure ER, and WEABs; and (**b**) time to the bitumen’s viscosity of WEABs at 120 °C.

The initial viscosity of epoxy resin at 120 °C (82 mPa∙s) is far lower than that of the bitumen (1120 mPa∙s), as depicted in Figure 2. Therefore, epoxy resin declines the initial viscosity of the neat bitumen (Figure 2a). Moreover, the initial viscosity of WEABs decreases in the epoxy resin concentration. The viscosity of the epoxy resin and WEABs increases with time because of chemical reactions between the epoxy oligomers and the curing agents as mentioned previously (Figure 1). After 20 min, WEABs have higher viscosity than neat bitumen. Furthermore, the time to the bitumen’s viscosity increases with the ER concentration (Figure 2b). It is worth mentioning that the reaction rate of pure ER is extremely slow. After curing for 120 min at 120 °C, pure ER’s viscosity only increases from 82 mPa∙s to 142 mPa∙s. Furthermore, pure ER was not completely cured after 4 h at 120 °C. For WEAB containing 90% ER, the viscosity increases from 107 mPa∙s to 721 mPa∙s at 120 min, which is lower than the bitumen’s viscosity (Figure 1b).

From the perspective of the pure ER, bitumen accelerates the cure reaction of ER since all the viscosities of WEABs are higher than that of the pure ER (Figure 1). Moreover, the acceleration effect boosts with the binder loading. Therefore, the viscosity of WEABs increases with the bitumen concentration. A similar trend was also reported in HEAB systems [24]. At the 50% bitumen concentration, the viscosity–time curve of WEAB shows the sharpest transition, indicating the fastest cure reaction of ER and the shortest allowable construction time of WEAB (Figure 1b). However, with the continuous increase in the binder concentration, the viscosity of WEABs lowers gradually (Figure 2). The catalytic effect of the binder can be attributed to the trace metal elements in bitumen. It is known that bitumen contains trace metals, such as iron, copper, nickel, and zinc, in the form of metallic oxides, salts, or porphyrin structures [28,29]. These metal salts and organo-transition metal complexes catalyze the chemical reactions of epoxy resin, especially at a low cure reaction rate [30,31]. Additionally, the catalytic effect depends on the phase-separated morphology of WEABs as will be discussed in Section 3.7.

### 3.2. Glass Transition Temperature

Measurement of T_g_ is a general method to judge the miscibility of polymer blends [32]. Polymer blends with a single T_g_ indicate that individual components are miscible and two T_g_s represent an immiscible (phase-separated) blend system. The main experimental techniques used to measure T_g_ of polymer blends involve DSC and DMA [33,34].

#### 3.2.1. Differential Scanning Calorimetry

**Figure 3:**
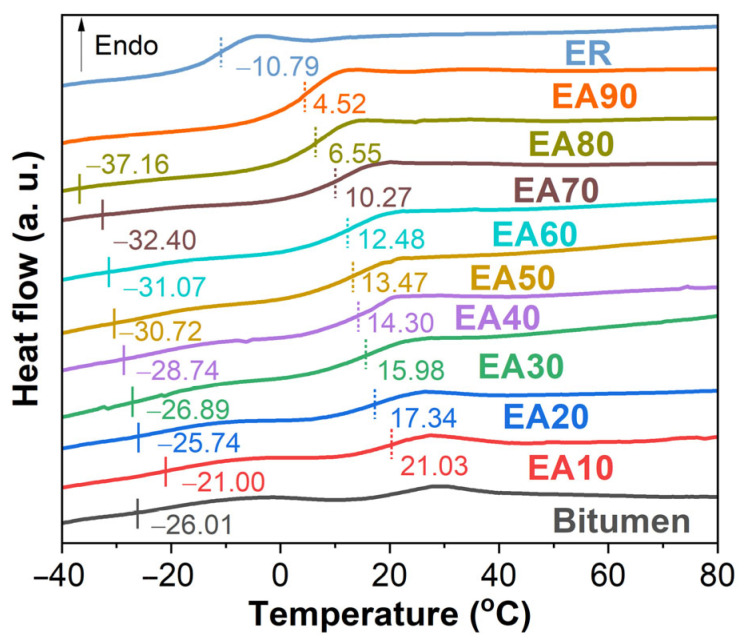
DSC curves of the bitumen, epoxy resin, and WEABs.

DSC thermograms of the pure ER, WEABs, and the neat bitumen are presented in Figure 3. Both the pure ER and bitumen exhibit a single T_g_, which are −10.79 and −26.01 °C, respectively. Moreover, EA90 also has a single T_g_ (4.52 °C), indicating that cured epoxy resin and bitumen are miscible in the WEAB with 90% ER. However, with the increase in binder concentration, two T_g_s appear, indicating the immiscibility between cured epoxy resin and the binder. Additionally, the T_g_s of both cured ER and bitumen increase with the binder concentration. In a word, the presence of bitumen increases the T_g_ of pure ER.

#### 3.2.2. Dynamic Mechanical Analysis

**Figure 4:**
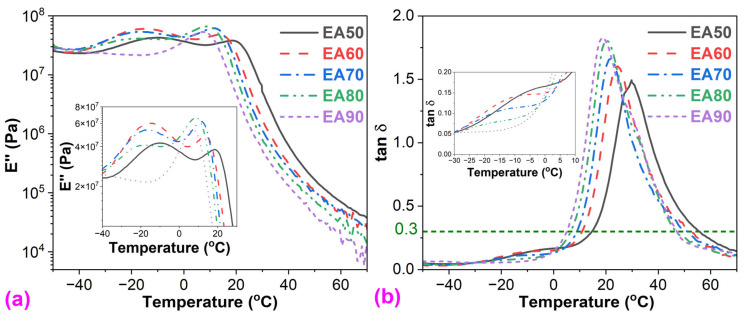
(**a**) *E″*-temperature; and (**b**) tan δ-temperature curves of WEABs.

In DMA, T_g_ can be obtained from the peak temperatures of *E″* (loss modulus)- and tan δ (damping factor or loss factor,)-temperature curves [35]. As shown in Figure 4, except for the WEAB containing 90% ER, two peaks for the T_g_s of both bitumen and cured ER appear in all *E″*— and tan δ—temperature curves, indicating the immiscibility between cured epoxy resin and bitumen. Table 2 summarizes the T_g_s of bitumen and epoxy resin of WEABs. Similar to DSC results, the T_g_s of both bitumen and cured epoxy resin decrease with the ER concentration.

As is known, the T_g_ of thermosetting polymers is highly correlated with their crosslinking density (*CD*), which can be obtained from Equation (1) on the basis of the elasticity theory [36]:

(3) $$CD=\frac{{E^{\prime }}_{T}^{\;\;}}{3RT},$$

where *T* is the temperature at the rubbery state, which equals T_g_ + 40 K. *R* is the gas constant. ${E^{\prime }}_{T}^{\;\;}$ is the storage modulus at *T*. The crosslinking density of WEABs decreases with the ER concentration as depicted in Table 2. In this case, with the increase in ER concentration, the T_g_s of both bitumen and cured ER decrease.

### 3.3. Damping Properties

Epoxy asphalt has excellent damping properties since both bitumen and epoxy resin are good viscoelastic damping materials for reducing vibration and noise [37]. Generally, three damping parameters are utilized to determine the damping ability of viscoelastic damping materials [38,39]: (1) the maximum value of tan δ [(tan δ)_max_]; (2) the area under the tan δ–temperature curve (*A*_t_). The greater these two values, the better the damping properties; (3) the effective damping temperature range at tan δ > 0.3 (Δ*T*). The wider the *A*_t_, the better the damping ability. 

As presented in Table 3, all damping parameters of WEABs increase with the ER concentration, indicating that the damping properties of WEABs are enhanced with the ER concentration. The damping ability indicates how the material loses energy efficiently through molecular rearrangement and internal friction [40,41]. From the viscoelasticity point of view, the damping ability of thermosetting polymer blends highly depends on both the viscous behavior and crosslinking density of the material. Furthermore, viscous behavior and crosslinking density have opposite effects on the damping ability of polymer blends. Conventional epoxies behave more like elastic polymers with a very weak viscous response. However, the viscous behavior of the pure ER used for the preparation of WEABs in this research is high, which is viscous even after curing for 4 h. That is to say, the pure ER has a high damping ability. With the addition of bitumen, the cure reaction of ER accelerates and higher crosslinking density is obtained (Table 2). Therefore, the damping ability of WEABs decreases with the bitumen concentration. It is important to mention that the damping ability of WEABs containing only six ER concentrations was studied in this research since samples with other concentrations are too soft to be measured by the DMA instrument with the tension mode. However, the damping ability of other samples can also be predicted. As already mentioned, bitumen has a good damping ability. WEABs with less than 50% ER concentrations can also have good damping properties due to their lower crosslinking density caused by bitumen as the continuous phase, as discussed in Section 3.7.

### 3.4. Storage Modulus

**Figure 5:**
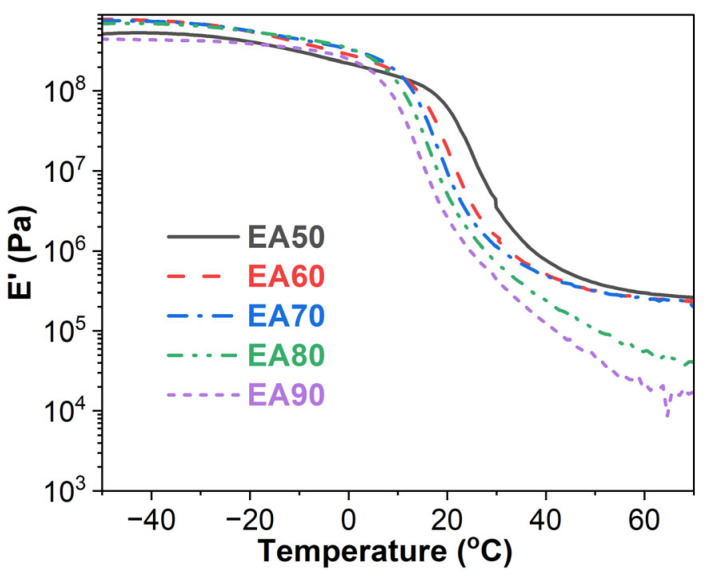
Storage modulus versus the temperature curves of WEABs.

Figure 5 shows the storage modulus vs. temperature of WEABs. During the glassy stage, the storage modulus of WEABs decreases with the ER concentration except for the 50% concentration, indicating the stiffness of WEABs decreases with the ER concentration. However, an opposite trend is shown during the glass transition and rubbery stage. 

Storage modulus can be converted to shear storage modulus (*G′*):

(4) $$E^{\prime }=2(1+\nu )G^{\prime }$$

where *ν* is the Poisson’s ratio (0.3–0.5) at the glassy and rubbery stage [42]. Cong and co-workers revealed that the shear storage modulus of epoxy asphalts boosts with the ER concentration (20–50%) at 60 °C [43]. However, in this work, WEABs exhibit a contrary trend with 50–90% ER concentrations at the rubbery stage.

### 3.5. Cole–Cole Plots

**Figure 6:**
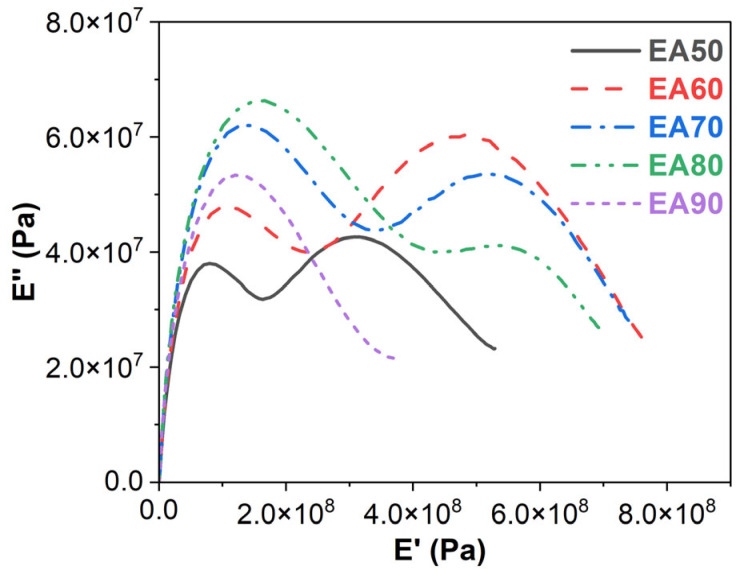
Cole–Cole plots of WEABs.

The Cole–Cole plots (*E″* vs. *E′*) of WEABs are shown in Figure 6. Except for the 90% concentration, all WEABs show two semicircles: the lower *E′* for bitumen and the high *E′* for cured epoxy resin, which indicate the heterogeneity of WEABs [44]. Furthermore, the E’s of both bitumen and cured epoxy resin shift to a higher modulus with the increase in ER loadings. For the WEAB containing 90% ER, only one semicircle shows in the lower modulus region, indicating that bitumen and epoxy resin are miscible at this ER concentration, as already mentioned.

### 3.6. Mechanical Properties

**Figure 7:**
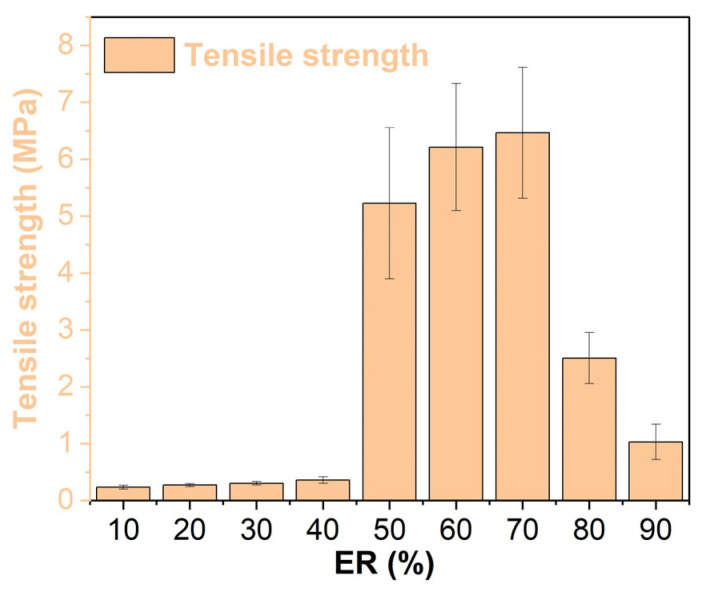
Tensile strength of WEABs.

The tensile strength of WEABs increases with the increase in ER concentrations (Figure 7). It is important to note that there is a sharp increment in tensile strength at a 50% ER concentration. The tensile strength of EA50 is more than 14-fold that of EA40. The tremendous change in the tensile strength of WEABs is caused by phase inversion. In other words, the continuous phase of WEAB converts from weak bitumen to strong epoxy resin, as discussed in Section 3.7 [27]. A maximal tensile strength value appears at a 70% ER concentration. After that, the tensile strength decreases, whose value is higher than that of EA40. Contrary to WEABs, the tensile strength of HEABs increases with the ER concentration [24]. A sudden boost of the tensile strength occurs at the 40% ER concentration.

**Figure 8:**
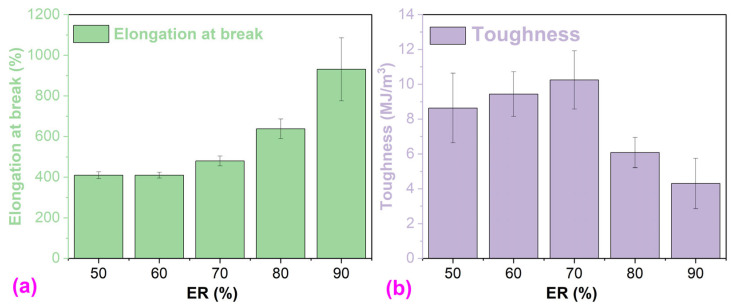
(**a**) Elongation at break and (**b**) toughness of WEABs.

With the increase in ER concentration, the elongation at break of WEABs increases (Figure 8a). However, the elongation at break of HEABs shows an opposite trend [24]. The area under the stress–strain curve presents the tensile toughness of a polymer [45,46]. Similarly to tensile strength, the tensile toughness of WEABs exhibits a maximal value at the 70% ER concentration. Afterward, the tensile toughness decreases with the ER concentration.

### 3.7. Phase-Separated Morphology

**Figure 9:**
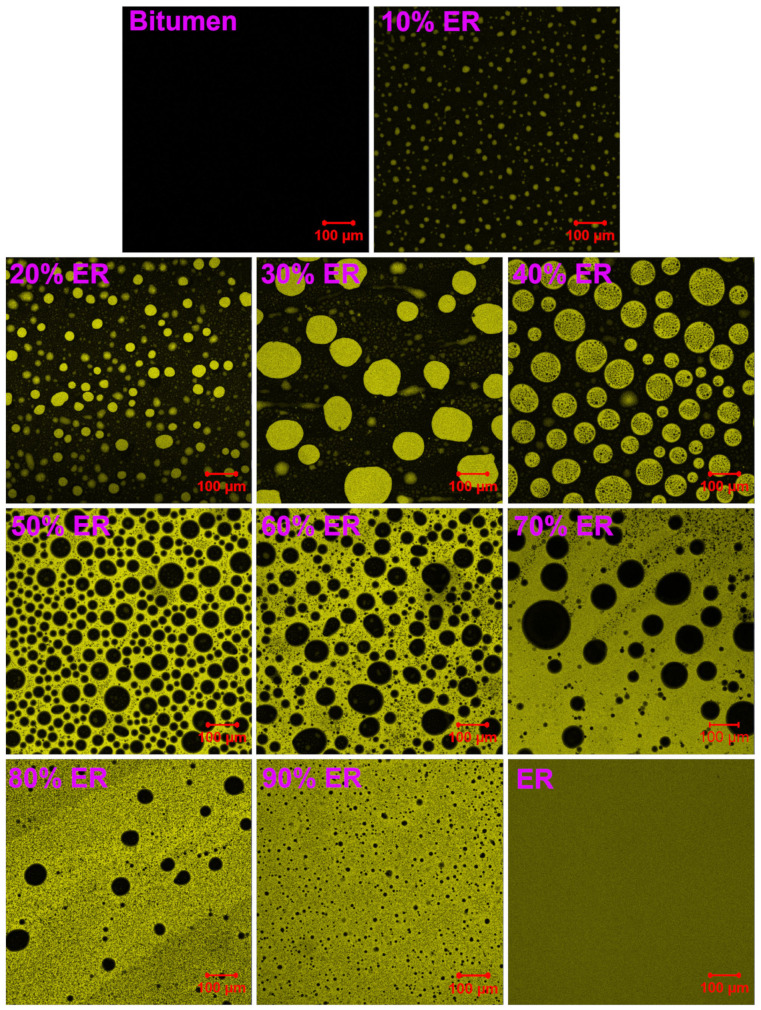
LSCM images with ×100 magnification of bitumen, cured WEABs, and pure ER.

Figure 9 shows the confocal microscopy micrographs of the neat bitumen, WEABs and pure epoxy resin. For the neat bitumen and pure epoxy resin, the surface is smooth. Phase-separated microstructures form in their blends (WEABs) because of the incompatibility between cured epoxy resin and bitumen, as discussed previously. Depending on the ER concentration, WEABs show two distinct phase-separated microstructures. In the case of WEABs with 10–40% ER, spherical epoxy particles act as the discontinuous phase. However, for WEABs with 50–90% ER, spherical bitumen particles become the discontinuous phase. That is to say, phase inversion takes place after 50% ER concentration and the discontinuous epoxy phase of WEABs converts into the continuous phase at this concentration. After phase inversion, the size of discontinuous bitumen particles first increases and a maximum size appears at 70% ER. Afterwards, the size of bitumen particles decreases.

Phase inversion causes great changes in the viscosity and tensile strength, as already mentioned. The continuous epoxy phase results in a dramatic increase in viscosity shortly after the mixing of components of WEABs due to the catalytic effect of bitumen, as shown in Figure 1. Moreover, the increment trend decreases with the ER concentration. In other words, the optimal bitumen concentration for the acceleration effect on the chemical reactions of epoxy resin is 50%. For mechanical properties, cured epoxy resin has a much higher mechanical performance than bitumen. Thus, after phase inversion, the WEAB with the continuous epoxy phase has a significantly higher tensile strength than the one with the continuous bitumen phase (Figure 7). 

It is of importance to note that WEAB containing 90% ER shows phase-separated morphology. However, as discussed previously, the results of T_g_ (Figure 3 and Figure 4) and Cole–Cole plots (Figure 6) show the contradictory result of bitumen and cured epoxy resin in this binder being miscible, and thus exhibiting structures of several nanometers, which are invisible in the LSCM image [47]. Thus, the use of the T_g_ and Cole–Cole plots to ascertain the miscibility of the polymer blend is not always universally accepted.

**Figure 10:**
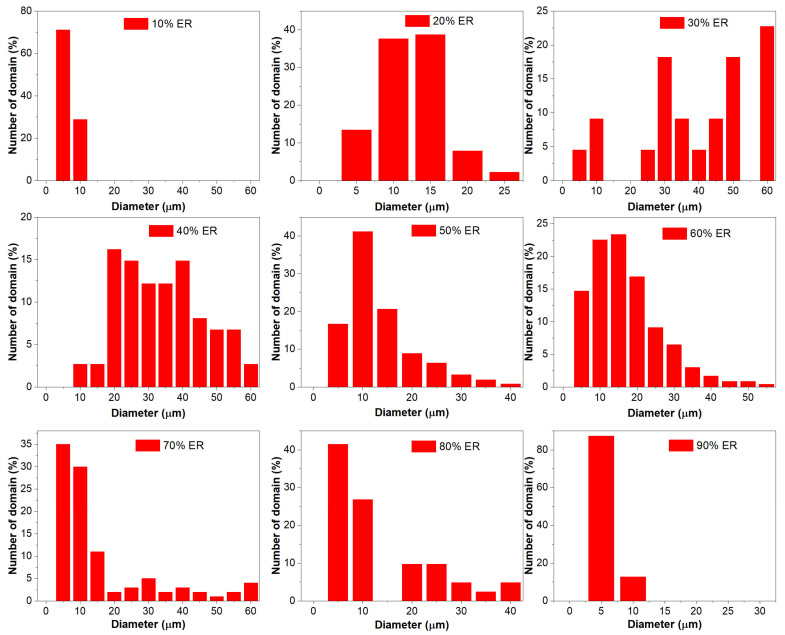
Particle size distribution of discontinuous epoxy or bitumen domains for WEABs.

Figure 10 illustrates the particle size distribution of discontinuous epoxy or bitumen domains in WEABs. For discontinuous epoxy domains in WEABs containing 10–40% ER, the particle size of large epoxy domains expands from 10 to 60 μm. WEAB with 10% ER shows a narrower particle size distribution, which concentrates at 5–10 μm. With the increase in ER concentration, the size of epoxy particles distributes in a larger range. Most particle sizes of WEAB with 20% ER are in the range of 10–15 μm, whereas the size of epoxy domains distributes randomly in WEABs with 30% and 40% ER. After phase inversion, the particle size distribution of discontinuous bitumen domains shows an opposite trend. 

Table 4 summarizes the average diameters of epoxy or bitumen particles and *PDI* (*d*_w_/*d*_n_) in WEABs. For discontinuous epoxy particles, both the average diameters and *PDI*s of WEABs with 10–30% ER increase with the ER concentration, while these parameters of WEAB with 40% ER show a slight decline compared to those of WEAB with 30% ER, indicating that the dispersion of epoxy particles in WEABs with 10–30% ER becomes randomly. However, a further increase in ER concentration results in a more uniform dispersion of epoxy particles compared to WEABs with 20% and 30% ER. After phase inversion, the average diameters of bitumen domains for WEABs containing 50% and 60% ER increase with the ER concentration, while a contrary trend appears after the 60% ER concentration. The value of *PDI* of WEABs with 50–70% ER increases with the ER concentration, whereas the *PDI*s of WEABs with 80% and 90% ER exhibit an opposite trend, indicating that the dispersion of bitumen particles becomes random and then uniform with the increase in the ER concentration.

It is worth noting that, except for the 20% ER concentration, discontinuous bitumen particles show smaller average diameters than discontinuous epoxy particles at the same bitumen and epoxy concentrations due to the existence of continuous and discontinuous epoxy phases in WEABs. That is to say, in comparison to the continuous bitumen phase, the continuous epoxy one confines the coalescence of bitumen particles and thus results in smaller bitumen particle size. However, *PDI* shows a contrary trend, indicating that free epoxy particles in the continuous bitumen phase tend to form a more uniform dispersion compared to the confined bitumen particles in the continuous epoxy phase.

## 4. Conclusions

This work investigated the properties and phase-separated morphology of WEABs containing various epoxy resin concentrations (10–90%). Particularly, the impact of phase inversion on the properties and microstructures of WEABs was emphasized. 

When the ER concentration is lower than 50%, epoxy particles disperse in the continuous bitumen phase of WEABs. After 50% ER, the continuous and discontinuous phases in WEABs invert. Bitumen becomes the discontinuous phase; in turn, epoxy resin turns to the continuous phase. The initial viscosity of WEABs decreases with the ER concentration due to the lower viscosity of ER in comparison to that of bitumen. However, except for the 90% ER, the time to the viscosity of bitumen increases with the ER concentration. Before phase inversion (10–40% ER), the viscosity of WEABs increases slowly with time. However, after phase inversion, the viscosity of WEABs with 50–80% ER increases sharply, but the increase tendency declines with the ER concentration. At 90% ER, the viscosity of WEAB increases too slowly to exceed the bitumen’s viscosity after a 150 min curing at 120 °C. Except for the 90% concentration, all WEABs have two T_g_s for both bitumen and cured epoxy resin, indicating the incompatibility between bitumen and cured epoxy resin. Furthermore, both T_g_s of bitumen and epoxy resin increase with the ER concentration. For WEABs with continuous epoxy phases, the crosslinking density decreases with the ER concentration, while damping properties exhibit an opposite trend. The tensile strength of WEABs with 10–70% increases with the ER concentration. Moreover, a sharp increase occurs at 50% ER because of the occurrence of phase inversion. After 70% ER, the tensile strength of WEABs decreases with the ER concentration. For WEABs with continuous epoxy phases, the elongation at break increases with the ER concentration. The toughness of WEABs with 50–70% ER increases with the ER concentration. After 70% ER, the toughness shows an opposite trend. 

In a word, from the perspective of mechanical performance, the WEAB with 50% epoxy resin is optimal for further research and practical application. Furthermore, since phase inversion shows an important impact on the properties of epoxy asphalt binders, it is of great interest to study the performance and phase-separated microstructures of WEABs in the vicinity of phase inversion.

## Figures and Tables

**Table 1:** Properties of bitumen.

Property	Standard	Value
Penetration (25 °C, 0.1 mm)	ASTM D5-06	73
Ductility (10 °C, cm)	ASTM D113-07	15.8
Softening point (°C)	ASTM D36-06	48.2
Viscosity (120 °C, mPa·s)	ASTM D4402-06	173
Saturates (%)	ASTM D4124-09	20.0
Aromatics (%)		31.5
Resins (%)		37.1
Asphaltenes (%)		6.8

**Table 2:** Glass transition temperatures and crosslinking densities of WEABs obtained from DMA.

ER (%)	*CD* (mol/m^3^)	T_g_ of Bitumen (°C)		T_g_ of Cured Epoxy (°C)		
		*E″*	tan δ	*E″*	tan δ	
50	30.8	−10.6	−6.7	18.5	29.8	
60	28.3	−15.6	−11.5	13.4	24.5	
70	28.1	−16.4	−15.5	11.8	22.1	
80	6.8	−18.9	−20.6	8.4	20.4	
90	3.4	−	−	7.3	18.5	

**Table 3:** Damping parameters of WEABs.

ER (%)	(tan δ)_max_	Δ*T* (°C)	*A*_t_ (K)
50	1.49	40.9 (14.5–55.4)	45.4
60	1.60	41.9 (10.8–52.7)	46.2
70	1.66	44.5 (8.5–53.0)	48.0
80	1.81	57.2 (6.8–64.0)	59.9
90	1.83	64.4 (5.0–69.4)	69.2

**Table 4:** Average diameters of discontinuous epoxy or bitumen particles and *PDI*s in WEABs.

ER (wt%)	*d*_n_ (μm)	*d*_w_ (μm)	*PDI*
10	6.6 ± 0.5	6.8 ± 0.4	1.03
20	13.1 ± 0.9	15.2 ± 1.1	1.16
30	39.3 ± 2.7	49.8 ± 2.3	1.27
40	32.3 ± 1.4	36.8 ± 1.8	1.14
50	15.4 ± 1.4	19.6 ± 1.7	1.27
60	17.1 ± 0.5	22.7 ± 0.9	1.33
70	17.2 ± 0.5	35.6 ± 3.1	2.06
80	14.0 ± 0.9	21.3 ± 0.2	1.52
90	6.2 ± 0.1	6.4 ± 0.2	1.04

## Data Availability

All data are available in the manuscript.
